# Genomic characterization of the 2019 novel human-pathogenic coronavirus isolated from a patient with atypical pneumonia after visiting Wuhan

**DOI:** 10.1080/22221751.2020.1719902

**Published:** 2020-01-28

**Authors:** Jasper Fuk-Woo Chan, Kin-Hang Kok, Zheng Zhu, Hin Chu, Kelvin Kai-Wang To, Shuofeng Yuan, Kwok-Yung Yuen

**Affiliations:** a State Key Laboratory of Emerging Infectious Diseases, The University of Hong Kong, Pokfulam, Hong Kong Special Administrative Region, China; b Department of Clinical Microbiology and Infection Control, The University of Hong Kong-Shenzhen Hospital, Shenzhen, Guangdong, People's Republic of China; c Department of Microbiology, Li Ka Shing Faculty of Medicine, The University of Hong Kong, Pokfulam, Hong Kong Special Administrative Region, China; d Carol Yu Centre for Infection, Li Ka Shing Faculty of Medicine, The University of Hong Kong, Pokfulam, Hong Kong Special Administrative Region, China

**Keywords:** Coronavirus, Wuhan, SARS, emerging, genome, respiratory, virus, bioinformatics

## Abstract

A mysterious outbreak of atypical pneumonia in late 2019 was traced to a seafood wholesale market in Wuhan of China. Within a few weeks, a novel coronavirus tentatively named as 2019 novel coronavirus (2019-nCoV) was announced by the World Health Organization. We performed bioinformatics analysis on a virus genome from a patient with 2019-nCoV infection and compared it with other related coronavirus genomes. Overall, the genome of 2019-nCoV has 89% nucleotide identity with bat SARS-like-CoVZXC21 and 82% with that of human SARS-CoV. The phylogenetic trees of their orf1a/b, Spike, Envelope, Membrane and Nucleoprotein also clustered closely with those of the bat, civet and human SARS coronaviruses. However, the external subdomain of Spike’s receptor binding domain of 2019-nCoV shares only 40% amino acid identity with other SARS-related coronaviruses. Remarkably, its orf3b encodes a completely novel short protein. Furthermore, its new orf8 likely encodes a secreted protein with an alpha-helix, following with a beta-sheet(s) containing six strands. Learning from the roles of civet in SARS and camel in MERS, hunting for the animal source of 2019-nCoV and its more ancestral virus would be important for understanding the origin and evolution of this novel lineage B *betacoronavirus*. These findings provide the basis for starting further studies on the pathogenesis, and optimizing the design of diagnostic, antiviral and vaccination strategies for this emerging infection.

## Introduction

Coronaviruses (CoVs) are enveloped, positive-sense, single-stranded RNA viruses that belong to the subfamily *Coronavirinae*, family *Coronavirdiae*, order *Nidovirales*. There are four genera of CoVs, namely, *Alphacoronavirus* (αCoV), *Betacoronavirus* (βCoV), *Deltacoronavirus* (δCoV), and *Gammacoronavirus* (γCoV) [1]. Evolutionary analyses have shown that bats and rodents are the gene sources of most αCoVs and βCoVs, while avian species are the gene sources of most δCoVs and γCoVs. CoVs have repeatedly crossed species barriers and some have emerged as important human pathogens. The best-known examples include severe acute respiratory syndrome CoV (SARS-CoV) which emerged in China in 2002–2003 to cause a large-scale epidemic with about 8000 infections and 800 deaths, and Middle East respiratory syndrome CoV (MERS-CoV) which has caused a persistent epidemic in the Arabian Peninsula since 2012 [2,3]. In both of these epidemics, these viruses have likely originated from bats and then jumped into another amplification mammalian host [the Himalayan palm civet (*Paguma larvata*) for SARS-CoV and the dromedary camel (*Camelus dromedarius*) for MERS-CoV] before crossing species barriers to infect humans.

Prior to December 2019, 6 CoVs were known to infect human, including 2 αCoV (HCoV-229E and HKU-NL63) and 4 βCoV (HCoV-OC43 [lineage A], HCoV-HKU1 [lineage A], SARS-CoV [lineage B] and MERS-CoV [lineage C]). The βCoV lineage A HCoV-OC43 and HCoV-HKU1 usually cause self-limiting upper respiratory infections in immunocompetent hosts and occasionally lower respiratory tract infections in immunocompromised hosts and elderly [4]. In contrast, SARS-CoV (lineage B βCoV) and MERS-CoV (lineage C βCoV) may cause severe lower respiratory tract infection with acute respiratory distress syndrome and extrapulmonary manifestations, such as diarrhea, lymphopenia, deranged liver and renal function tests, and multiorgan dysfunction syndrome, among both immunocompetent and immunocompromised hosts with mortality rates of ∼10% and ∼35%, respectively [5,6]. On 31 December 2019, the World Health Organization (WHO) was informed of cases of pneumonia of unknown cause in Wuhan City, Hubei Province, China [7]. Subsequent virological testing showed that a novel CoV was detected in these patients. As of 16 January 2020, 43 patients have been diagnosed to have infection with this novel CoV, including two exported cases of mild pneumonia in Thailand and Japan [8,9]. The earliest date of symptom onset was 1 December 2019 [10]. The symptomatology of these patients included fever, malaise, dry cough, and dyspnea. Among 41 patients admitted to a designated hospital in Wuhan, 13 (32%) required intensive care and 6 (15%) died. All 41 patients had pneumonia with abnormal findings on chest computerized tomography scans [10].

We recently reported a familial cluster of 2019-nCoV infection in a Shenzhen family with travel history to Wuhan [11]. In the present study, we analyzed a 2019-nCoV complete genome from a patient in this familial cluster and compared it with the genomes of related β CoVs to provide insights into the potential source and control strategies.

## Materials and methods

### Viral sequences

The complete genome sequence of 2019-nCoV HKU-SZ-005b was available at GenBank (accession no. MN975262) (Table 1). The representative complete genomes of other related βCoVs strains collected from human or mammals were included for comparative analysis. These included strains collected from human, bats, and Himalayan palm civet between 2003 and 2018, with one 229E coronavirus strain as the outgroup.

**Table 1.** List of coronaviruses used in this study.

Accession number	Name displayed on the tree	Name of full-length genome	Year
AY274119	Human SARS-CoV Tor2 2003	SARS-related coronavirus isolate Tor2	2003
AY278488	Human SARS-CoV BJ01 2003	SARS coronavirus BJ01	2003
AY278491	SARS coronavirus HKU-39849 2003	SARS coronavirus HKU-39849 2003	2003
AY390556	Human SARS-CoV GZ02 2003	SARS coronavirus GZ02	2003
AY391777	Human CoV OC43 2003	Human coronavirus OC43	2003
AY515512	Paguma SARS CoV HC/SZ/61/03 2003	SARS coronavirus HC/SZ/61/03 (paguma SARS)	2018
EF065513	Bat CoV HKU9-1 2006	Bat coronavirus HKU9-1	2006
FJ588686	Bat SL-CoV Rs672 2006	Bat SARS CoV Rs672/2006	2006
KC881005	Bat SL-CoV RsSHC014 2013	Bat SARS-like coronavirus RsSHC014	2013
KC881006	Bat SL-CoV Rs3367 2013	Bat SARS-like coronavirus Rs3367	2013
KY417146	Bat SL-CoV Rs4231 2016	Bat SARS-like coronavirus isolate Rs4231	2016
KY417149	Bat SL-CoV Rs4255 2016	Bat SARS-like coronavirus isolate Rs4255	2016
MG772933	Bat SL-CoV ZC45 2018	Bat SARS-like coronavirus isolate bat-SL-CoVZC45	2018
MG772934	Bat SL-CoV ZXC21 2018	Bat SARS-like coronavirus isolate bat-SL-CoVZXC21	2018
MK211377	Bat CoV YN2018C 2018	Coronavirus BtRs-BetaCoV/YN2018C	2018
MK211378	Bat CoV YN2018D 2018	Coronavirus BtRs-BetaCoV/YN2018D^a^	2018
MN975262	HKU-SZ-005b	Human 2019-nCoV HKU-SZ-005b	2020
NC002645	Human CoV 229E 2000	Human coronavirus 229E	2000
NC006577	Human CoV HKU1 2004	Human coronavirus HKU1	2004
NC009019	Bat CoV HKU4-1 2006	Bat coronavirus HKU4-1	2006
NC009020	Bat CoV HKU5-1 2006	Bat coronavirus HKU5-1	2006
NC014470	Bat SARS-related CoV BM48-31 2009	Bat coronavirus BM48-31/BGR/2008	2008
NC019843	Human MERS-CoV 2012	Middle East respiratory syndrome coronavirus	2012

^a^One nucleotide was added within M gene to maintain the sequence in-frame.

### Genome characterization and phylogenetic analysis

Phylogenetic tree construction by the neighbour joining method was performed using MEGA X software, with bootstrap values being calculated from 1000 trees [12]. The percentage of replicate trees in which the associated taxa clustered together in the bootstrap test (1000 replicates) was shown next to the branches [13]. The tree was drawn to scale, with branch lengths in the same units as those of the evolutionary distances used to infer the phylogenetic tree. The evolutionary distances were computed using the Poisson correction method and were in the units of the number of amino acid substitutions per site [14]. All ambiguous positions were removed for each sequence pair (pairwise deletion option). Evolutionary analyses were conducted in MEGA X [15]. Multiple alignment was performed using CLUSTAL 2.1 and further visualized using BOXSHADE 3.21. Structural analysis of orf8 was performed using PSI-blast-based secondary structure PREDiction (PSIPRED) [16]. For the prediction of protein secondary structure including beta sheet, alpha helix, and coil, initial amino acid sequences were input and analysed using neural networking and its own algorithm. Predicted structures were visualized and highlighted on the BOXSHADE alignment. Prediction of transmembrane domains was performed using the TMHMM 2.0 server (http://www.cbs.dtu.dk/services/TMHMM/). Secondary structure prediction in the 5′-untranslated region (UTR) and 3′-UTR was performed using the RNAfold WebServer (http://rna.tbi.univie.ac.at/cgi-bin/RNAWebSuite/RNAfold.cgi) with minimum free energy (MFE) and partition function in Fold algorithms and basic options. The human SARS-CoV 5′- and 3′- UTR were used as references to adjust the prediction results.

## Results and discussion

### Genome organization

**Figure 1.**
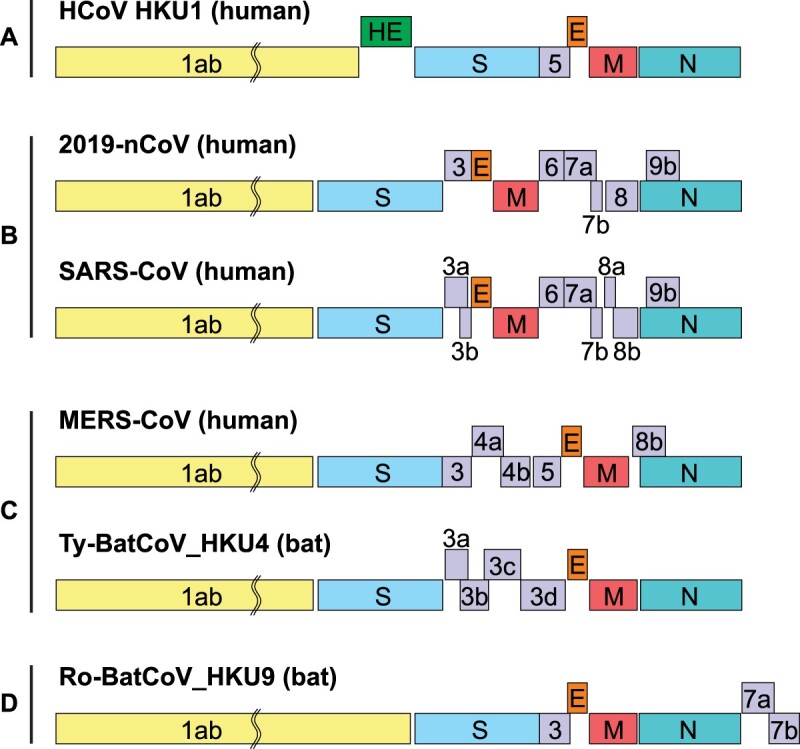
Betacoronavirus genome organization. The betacoronavirus genome comprises of the 5'-untranslated region (5'-UTR), open reading frame (orf) 1a/b (yellow box) encoding non-structural proteins (nsp) for replication, structural proteins including spike (blue box), envelop (orange box), membrane (red box), and nucleocapsid (cyan box) proteins, accessory proteins (purple boxes) such as orf 3, 6, 7a, 7b, 8 and 9b in the 2019-nCoV (HKU-SZ-005b) genome, and the 3'-untranslated region (3'-UTR). Examples of lineages A to D betacoronaviruses include human coronavirus (HCoV) HKU1 (lineage A), 2019-nCoV (HKU-SZ-005b) and SARS-CoV (lineage B), MERS-CoV and *Tylonycteris* bat CoV HKU4 (lineage C), and *Rousettus* bat CoV HKU9 (lineage D). The length of nsps and orfs are not drawn in scale.

The single-stranded RNA genome of the 2019-nCoV was 29891 nucleotides in size, encoding 9860 amino acids. The G + C content was 38%. Similar to other βCoVs, the 2019-nCoV genome contains two flanking untranslated regions (UTRs) and a single long open reading frame encoding a polyprotein. The 2019-nCoV genome is arranged in the order of 5′-replicase (orf1/ab)-structural proteins [Spike (S)-Envelope (E)-Membrane (M)-Nucleocapsid (N)]−3′ and lacks the hemagglutinin-esterase gene which is characteristically found in lineage A β-CoVs (Figure 1).

There are 12 putative, functional open reading frames (orfs) expressed from a nested set of 9 subgenomic mRNAs carrying a conserved leader sequence in the genome, 9 transcription-regulatory sequences, and 2 terminal untranslated regions. The 5′- and 3′-UTRs are 265 and 358 nucleotides long, respectively. The 5′- and 3 ′-UTR sequences of 2019-nCoV are similar to those of other βCoVs with nucleotide identities of ⩾83.6%. The large replicase polyproteins pp1a and pp1ab encoded by the partially overlapping 5′-terminal orf1a/b within the 5′ two-thirds of the genome is proteolytic cleaved into 16 putative non-structural proteins (nsps). These putative nsps included two viral cysteine proteases, namely, nsp3 (papain-like protease) and nsp5 (chymotrypsin-like, 3C-like, or main protease), nsp12 (RNA-dependent RNA polymerase [RdRp]), nsp13 (helicase), and other nsps which are likely involved in the transcription and replication of the virus (Table 2). There are no remarkable differences between the orfs and nsps of 2019-nCoV with those of SARS-CoV (Table 3). The major distinction between SARSr-CoV and SARS-CoV is in orf3b, Spike and orf8 but especially variable in Spike S1 and orf8 which were previously shown to be recombination hot spots.

**Table 2.** Putative functions and proteolytic cleavage sites of 16 nonstructural proteins in orf1a/b as predicted by bioinformatics.

NSP	Putative function/domain	Amino acid position	Putative cleave site
nsp1	suppress antiviral host response	M1 – G180	(LNGG'AYTR)
nsp2	unknown	A181 – G818	(LKGG'APTK)
nsp3	putative PL-pro domain	A819 – G2763	(LKGG'KIVN)
nsp4	complex with nsp3 and 6: DMV formation	K2764 – Q3263	(AVLQ'SGFR)
nsp5	3CL-pro domain	S3264 – Q3569	(VTFQ'SAVK)
nsp6	complex with nsp3 and 4: DMV formation	S3570 – Q3859	(ATVQ'SKMS)
nsp7	complex with nsp8: primase	S3860 – Q3942	(ATLQ'AIAS)
nsp8	complex with nsp7: primase	A3943 – Q4140	(VKLQ'NNEL)
nsp9	RNA/DNA binding activity	N4141 – Q4253	(VRLQ'AGNA)
nsp10	complex with nsp14: replication fidelity	A4254 – Q4392	(PMLQ'SADA)
nsp11	short peptide at the end of orf1a	S4393 – V4405	(end of orf1a)
nsp12	RNA-dependent RNA polymerase	S4393 – Q5324	(TVLQ'AVGA)
nsp13	helicase	A5325 – Q5925	(ATLQ'AENV)
nsp14	ExoN: 3′–5′ exonuclease	A5926 – Q6452	(TRLQ'SLEN)
nsp15	XendoU: poly(U)-specific endoribonuclease	S6453 – Q6798	(PKLQ'SSQA)
nsp16	2'-O-MT: 2'-O-ribose methyltransferase	S6799 – N7096	(end of orf1b)

**Table 3.** Amino acid identity between the 2019 novel coronavirus and bat SARS-like coronavirus or human SARS-CoV.

Amino acid identity (%)	2019-nCoV	2019-nCoV
	vs. bat-SL-CoVZXC21	vs. SARS-CoV
NSP1	96	84
NSP2	96	68
NSP3	93	76
NSP4	96	80
NSP5	99	96
NSP6	98	88
NSP7	99	99
NSP8	96	97
NSP9	96	97
NSP10	98	97
NSP11	85	85
NSP12	96	96
NSP13	99	100
NSP14	95	95
NSP15	88	89
NSP16	98	93
Spike	80	76
Orf3a	92	72
Orf3b	32	32
Envelope	100	95
Membrane	99	91
Orf6	94	69
Orf7a	89	85
Orf7b	93	81
Orf8/Orf8b	94	40
Nucleoprotein	94	94
Orf9b	73	73

#### Spike

**Figure 2.**
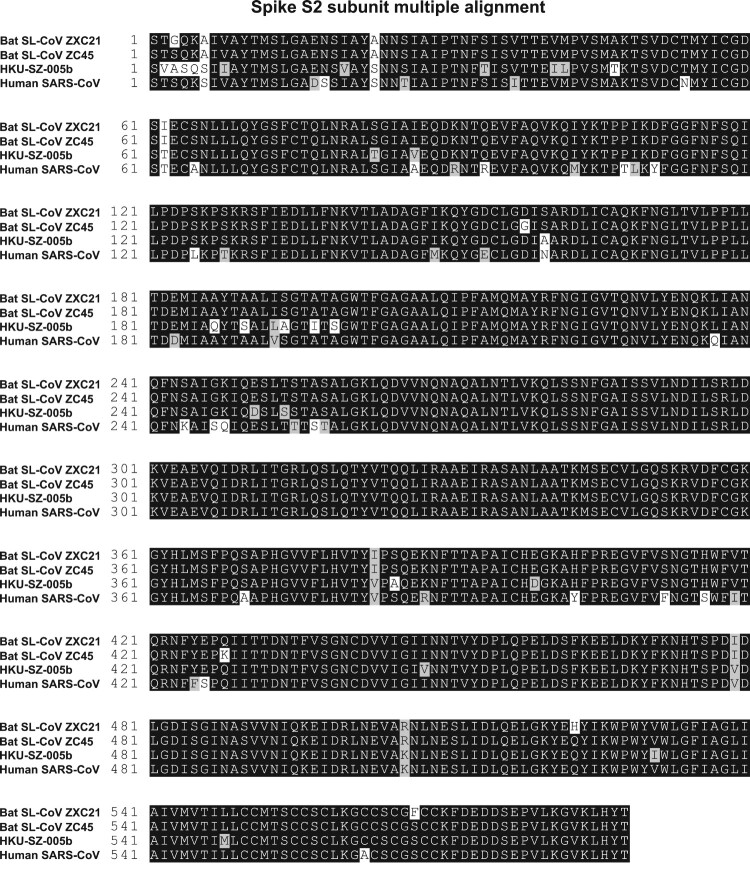
Comparison of protein sequences of Spike stalk S2 subunit. Multiple alignment of Spike S2 amino acid sequences of 2019-nCoV HKU-SZ-005b (accession number MN975262), bat SARS-like coronavirus isolates bat-SL-CoVZXC21 and bat-SL-CoVZXC45 (accession number MG772934.1 and MG772933.1, respectively) and human SARS coronavirus (accession number NC004718) was performed and displayed using CLUSTAL 2.1 and BOXSHADE 3.21 respectively. The black boxes represent the identity while the grey boxes represent the similarity of the four amino acid sequences.

Spike glycoprotein comprised of S1 and S2 subunits. The S1 subunit contains a signal peptide, followed by an N-terminal domain (NTD) and receptor-binding domain (RBD), while the S2 subunit contains conserved fusion peptide (FP), heptad repeat (HR) 1 and 2, transmembrane domain (TM), and cytoplasmic domain (CP). We found that the S2 subunit of 2019-nCoV is highly conserved and shares 99% identity with those of the two bat SARS-like CoVs (SL-CoV ZXC21 and ZC45) and human SARS-CoV (Figure 2). Thus the broad spectrum antiviral peptides against S2 would be an important preventive and treatment modality for testing in animal models before clinical trials [18]. Though the S1 subunit of 2019-nCoV shares around 70% identity to that of the two bat SARS-like CoVs and human SARS-CoV (Figure 3(A)), the core domain of RBD (excluding the external subdomain) are highly conserved (Figure 3(B)). Most of the amino acid differences of RBD are located in the external subdomain, which is responsible for the direct interaction with the host receptor. Further investigation of this soluble variable external subdomain region will reveal its receptor usage, interspecies transmission and pathogenesis. Unlike 2019-nCoV and human SARS-CoV, most known bat SARSr-CoVs have two stretches of deletions in the spike receptor binding domain (RBD) when compared with that of human SARS-CoV. But some Yunnan strains such as the WIV1 had no such deletions and can use human ACE2 as a cellular entry receptor. It is interesting to note that the two bat SARS-related coronavirus ZXC21 and ZC45, being closest to 2019-nCoV, can infect suckling rats and cause inflammation in the brain tissue, and pathological changes in lung & intestine. However, these two viruses could not be isolated in Vero E6 cells and were not investigated further. The two retained deletion sites in the Spike genes of ZXC21 and ZC45 may lessen their likelihood of jumping species barriers imposed by receptor specificity.

Figure 3.Comparison of protein sequences of A. Spike globular head S1, and B. S1 receptor-binding domain (RBD) subunit. Multiple alignment of Spike S1 amino acid sequences of 2019-nCoV HKU-SZ-005b (accession number MN975262), bat SARS-like coronavirus isolates bat-SL-CoVZXC21, bat-SL-CoVZXC45, bat-SL-CoV-YNLF_31C, bat-SL-CoV-YNLF_34C and bat SL-CoV HKU3-1 (accession number MG772934.1 and MG772933.1, KP886808, KP886809 and DQ022305, respectively), human SARS coronavirus GZ02 and Tor2 (accession number AY390556 and AY274119, respectively) and Paguma SARS-CoV (accession number AY515512) was performed and displayed using CLUSTAL 2.1 and BOXSHADE 3.21, respectively. The black background represents the identity while the grey background represents the similarity of the amino acid sequences. Orange box indicates the region of signal peptide, while green and blue boxes indicate the core domain and receptor binding domain respectively. Sequences of RBD, highlighted in (A) were used for comparison. External subdomain variable region of 2019-nCoV HKU-SZ-005b was predicted by comparison of amino acid similarity and published structural analysis [17]. Purple box indicates the external subdomain region.

**Figure:**
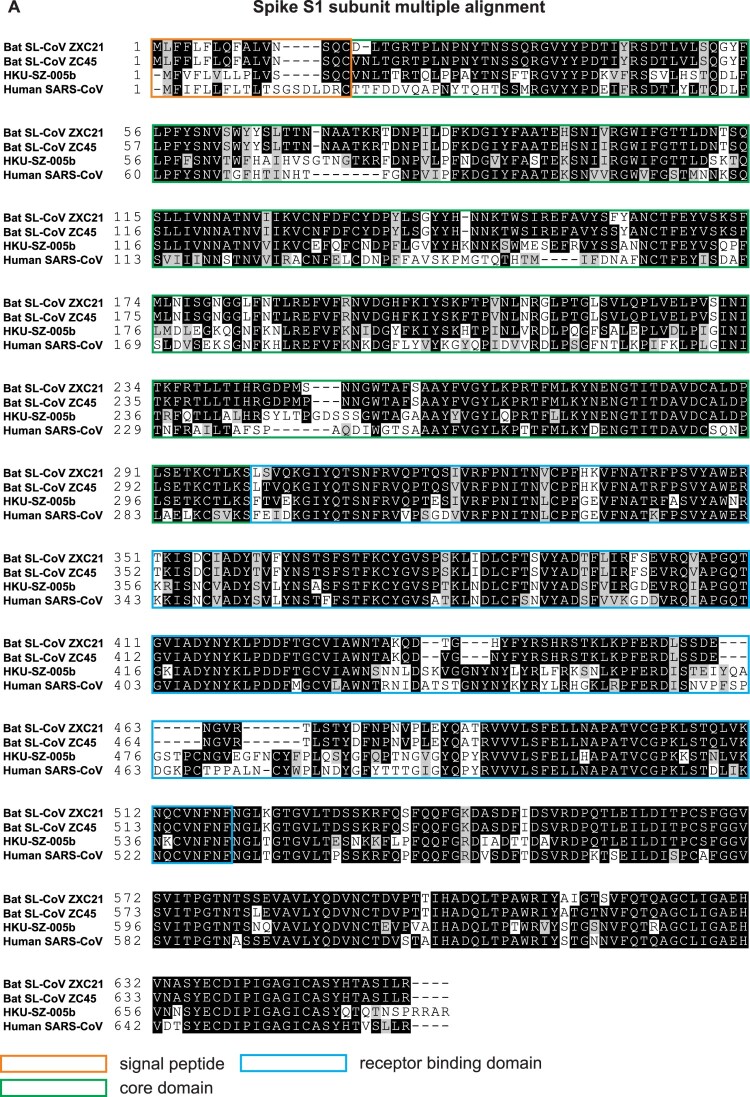

**Figure:**
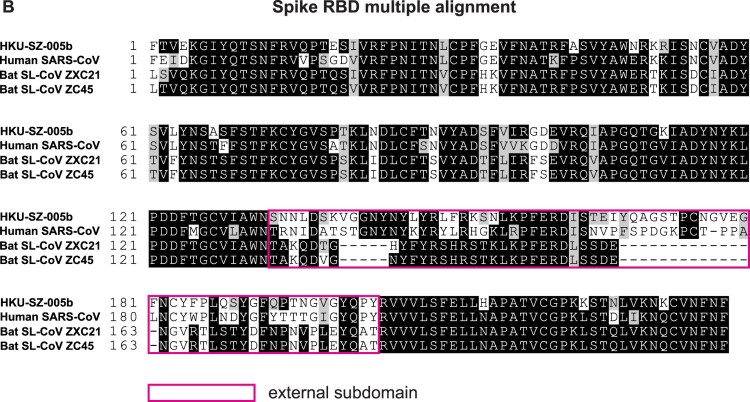

#### Orf3b

**Figure 4.**
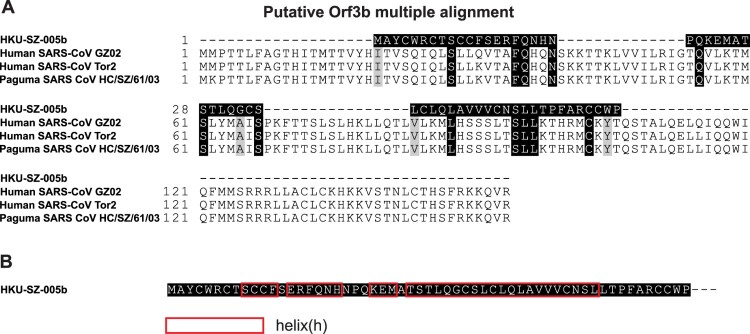
Analysis of orf3b. A. Multiple alignment of orf3b protein sequence between 2019-nCoV (HKU-SZ-005b), SARS-CoV and SARS-related CoV. B. A novel putative short protein found in orf3b.

A novel short putative protein with 4 helices and no homology to existing SARS-CoV or SARS-r-CoV protein was found within Orf3b (Figure 4). It is notable that SARS-CoV deletion mutants lacking orf3b replicate to levels similar to those of wild-type virus in several cell types [19], suggesting that orf3b is dispensable for viral replication in vitro. But orf3b may have a role in viral pathogenicity as Vero E6 but not 293T cells transfected with a construct expressing Orf3b underwent necrosis as early as 6 h after transfection and underwent simultaneous necrosis and apoptosis at later time points [20]. Orf3b was also shown to inhibit expression of IFN-β at synthesis and signalling [21]. Subsequently, orf3b homologues identified from three bat SARS-related-CoV strains were C-terminally truncated and lacked the C-terminal nucleus localization signal of SARS-CoV [22]. IFN antagonist activity analysis demonstrated that one SARS-related-CoV orf3b still possessed IFN antagonist and IRF3-modulating activities. These results indicated that different orf3b proteins display different IFN antagonist activities and this function is independent of the protein's nuclear localization, suggesting a potential link between bat SARS-related-CoV orf3b function and pathogenesis. The importance of this new protein in 2019-nCoV will require further validation and study.

#### Orf8

**Figure 5.**
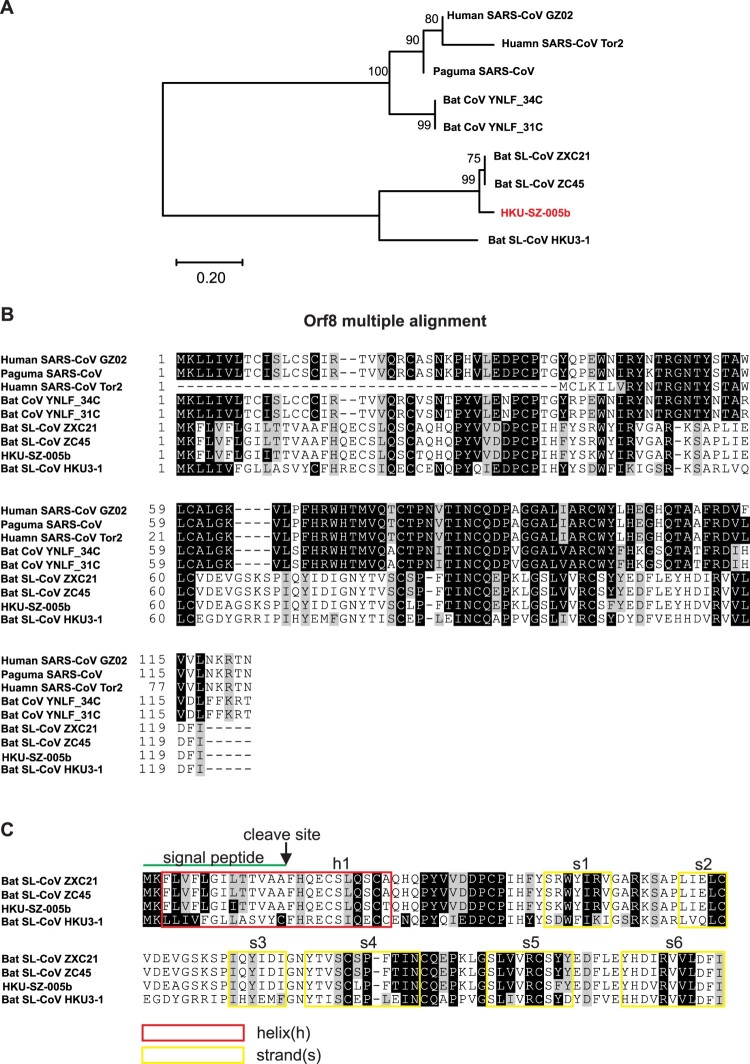
Analysis of orf8 to show novel putative protein. (A) Phylogenetic analysis of orf8 amino acid sequences of 2019-nCoV HKU-SZ-005b (accession number MN975262), bat SARS-like coronavirus isolates bat-SL-CoVZXC21 and bat-SL-CoVZXC45 (accession number MG772934.1 and MG772933.1, respectively) and human SARS coronavirus (accession number AY274119) was performed using the neighbour-joining method with bootstrap 1000. The evolutionary distances were calculated using the JTT matrix-based method. (B) Multiple alignment was performed and displayed using CLUSTAL 2.1 and BOXSHADE 3.21, respectively. The black background represents the identity while the grey background represents the similarity of the amino acid sequences. (C) Structural analysis of Orf8 was performed using PSI-blast-based secondary structure PREDiction (PSIPRED). Predicted helix structure (h) and strand (s) were boxed with red and yellow respectively.

orf8 is an accessory protein found in the *Betacoronavirus* lineage B coronaviruses. Human SARS-CoVs isolated from early-phase patients, all civet SARS-CoVs, and other bat SARS-related CoVs contain full-length orf8 [23]. However, a 29-nucleotide deletion, which causes the split of full length of orf8 into putative orf8a and orf8b, has been found in all SARS-CoV isolated from mid- and late- phase human patients [24]. In addition, we have previously identified two bat SARS-related-CoV (Bat-CoV YNLF_31C and YNLF_34C) and proposed that the original SARS-CoV full-length orf8 is acquired from these two bat SARS-related-CoV [25]. Since the SARS-CoV is the closest human pathogenic virus to the 2019-nCoV, we performed phylogenetic analysis and multiple alignments to investigate the orf8 amino acid sequences. The orf8 protein sequences used in the analysis derived from early phase SARS-CoV that includes full-length orf8 (human SARS-CoV GZ02), the mid- and late-phase SARS-CoV that includes the split orf8b (human SARS-CoV Tor2), civet SARS-CoV (paguma SARS-CoV), two bat SARS-related-CoV containing full-length orf8 (bat-CoV YNLF_31C and YNLF_34C), 2019-nCoV, the other two closest bat SARS-related-CoV to 2019-nCoV SL-CoV ZXC21 and ZC45), and bat SARS-related-CoV HKU3-1 (Figure 5(A)). As expected, orf8 derived from 2019-nCoV belongs to the group that includes the closest genome sequences of bat SARS-related-CoV ZXC21 and ZC45. Interestingly, the new 2019-nCoV orf8 is distant from the conserved orf8 or orf8b derived from human SARS-CoV or its related viruses derived from civet (paguma SARS-CoV) and bat (bat-CoV YNLF_31C and YNLF_34C). This new orf8 of 2019-nCoV does not contain known functional domain or motif. An aggregation motif VLVVL (amino acid 75–79) has been found in SARS-CoV orf8b (Figure 5(B)) which was shown to trigger intracellular stress pathways and activates NLRP3 inflammasomes [26], but this is absent in this novel orf8 of 2019-nCoV. Based on a secondary structure prediction, this novel orf8 has a high possibility to form a protein with an alpha-helix, following with a beta-sheet(s) containing six strands (Figure 5(C)).

#### Phylogenetic relationship among 2019-nCoV and other βCoVs

The genome of 2019-nCoV has overall 89% nucleotide identity with bat SARS-related-CoV SL-CoVZXC21 (MG772934.1), and 82% with human SARS-CoV BJ01 2003 (AY278488) and human SARS-CoV Tor2 (AY274119). The phylogenetic trees constructed using the amino acid sequences of orf1a/b and the 4 structural genes (S, E, M, and N) were shown (Figure 6(A–E)). For all these 5 genes, the 2019-nCoV was clustered with lineage B βCoVs. It was most closely related to the bat SARS-related CoVs ZXC21 and ZC45 found in Chinese horseshoe bats (*Rhinolopus sinicus*) collected from Zhoushan city, Zhejiang province, China between 2015 and 2017. Thus this novel coronavirus should belong to the genus *Betacoronavirus*, subgenus *Sabecovirus* (previously lineage 2b of Group 2 coronavirus). SARS-related coronaviruses have been found continuously especially in horseshoe bat species in the last 13 years. Between 2003 and 2018, 339 complete SARS-related coronavirus genomes have been sequenced, including 274 human SARS-CoV, 18 civet SARS coronavirus, and 47 bat SARS-related coronaviruses mainly from *Rhinolophus* bat species. Together, they formed a distinct subclade among other lineage B βCoVs. These results suggested that the 2019-nCoV might have also originated from bats. But we cannot ascertain whether another intermediate or amplification animal host infected by 2019-nCoV could be found in the epidemiological market, just as in the case of Paguma civets for SARS-CoV. Figure 6.Phylogenetic tree construction by the neighbour joining method was performed using MEGA X software, with bootstrap values being calculated from 1000 trees using amino acid sequences of (A) orf1ab polypeptide; (B) Spike glycoprotein; (C) Envelope protein; (D) Membrane protein; (E) Nucleoprotein.

**Figure:**
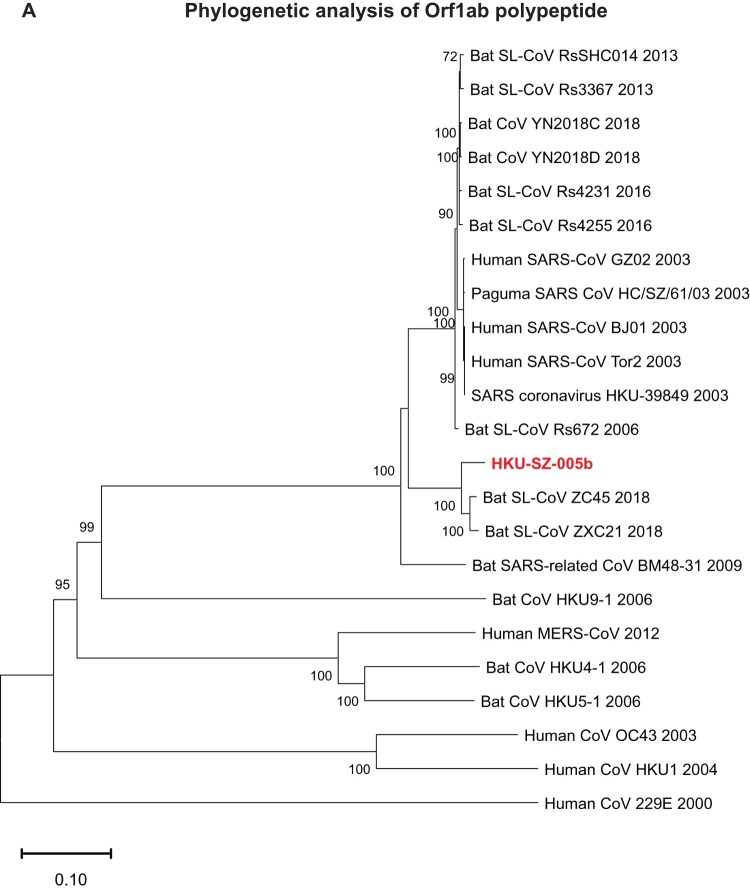

**Figure:**
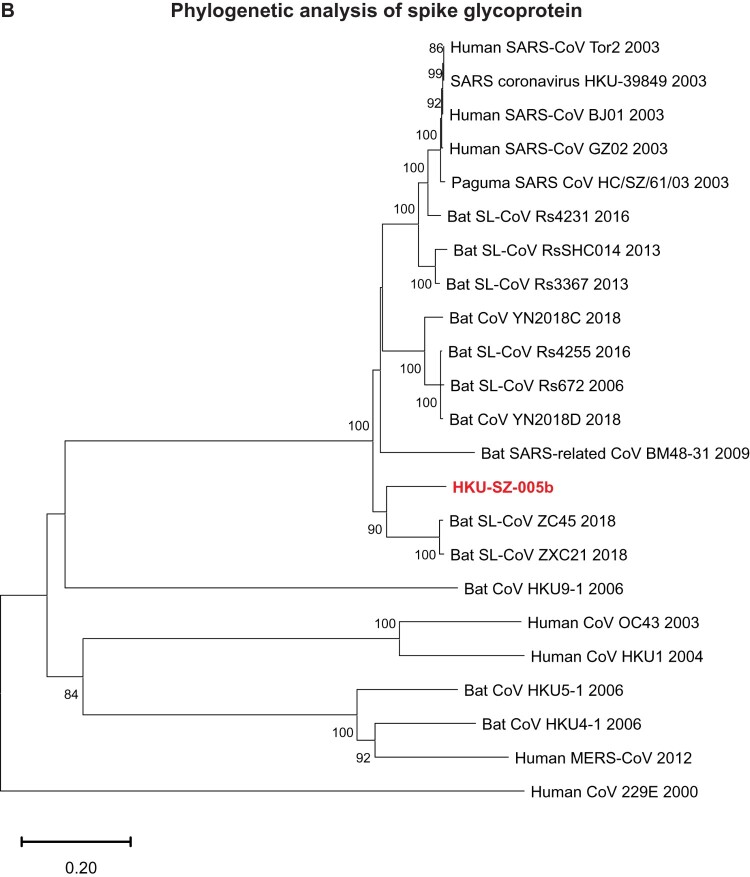

**Figure:**
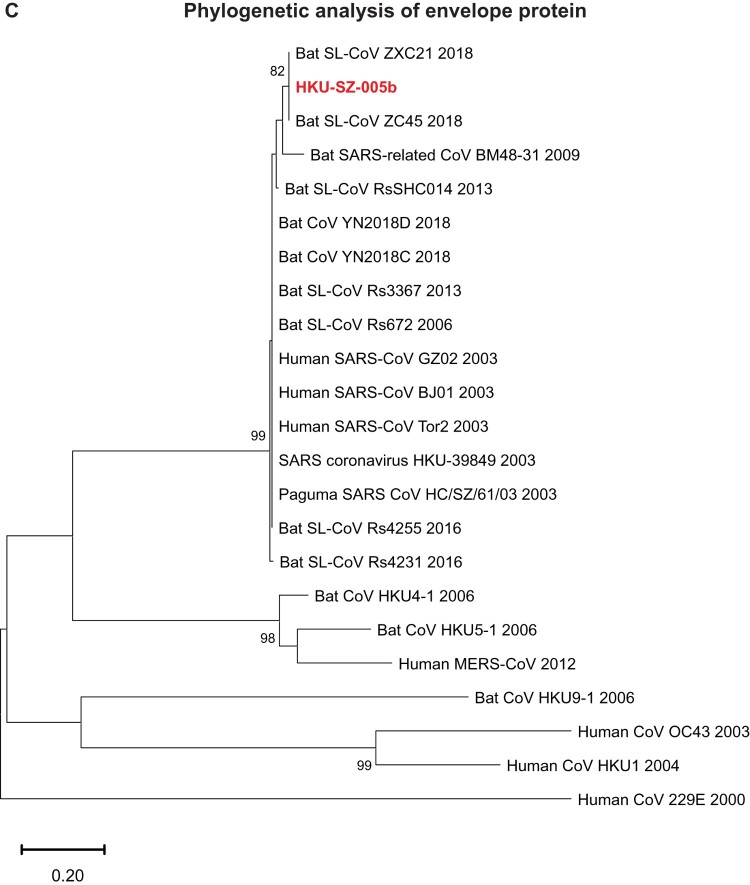

**Figure:**
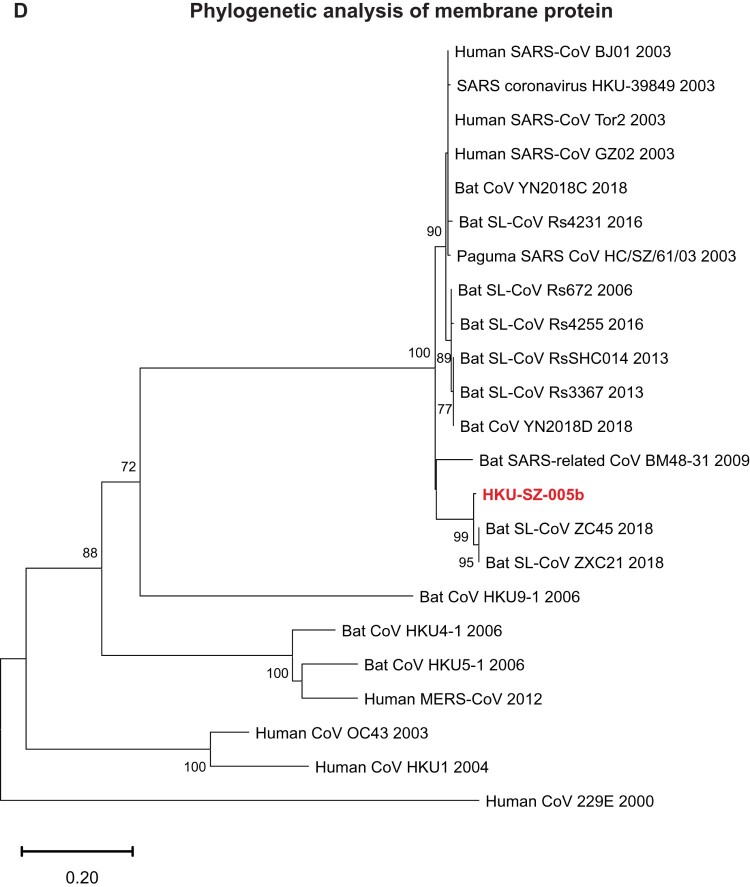

**Figure:**
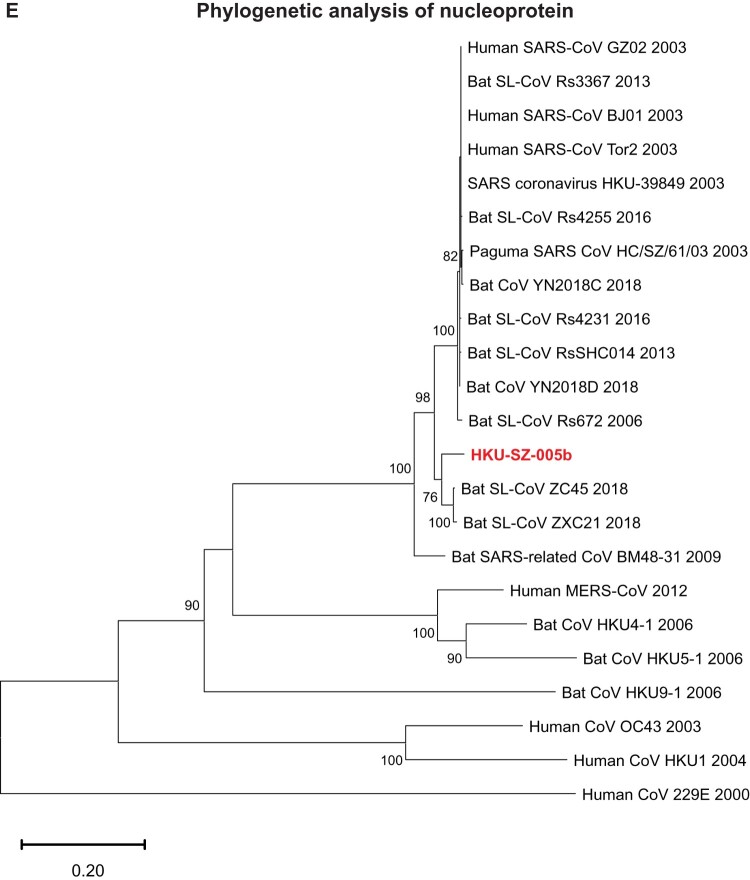

#### RNA secondary structures

As shown in Figure 7(A–C), the SARS-CoV 5′-UTR contains SL1, SL2, SL3, SL4, S5, SL5A, SL5B, SL5C, SL6, SL7, and SL8. The SL3 contains trans–cis motif [27]. The SL1, SL2, SL3, SL4, S5, SL5A, SL5B, and SL5C structures were similar among the 2019-nCoV, human SARS-CoV and the bat SARS-related ZC45. In the 2019-nCoV, part of the S5 found was inside the orf1a/b (marked in red), which was similar to SARS-CoV. In bat SARS-related CoV ZC45, the S5 was not found inside orf1a/b. The 2019-nCoV had the same SL6, SL7, and SL8 as SARS-CoV, and an additional stem loop. Bat SARS-related CoV ZC45 did not have the SARS-COV SL6-like stem loop. Instead, it possessed two other stem loops in this region. All three strains had similar SL7 and SL8. The bat SARS-like CoV ZC45 also had an additional stem loop between SL7 and SL8. Overall, the 5′-UTR of 2019-nCoV was more similar to that of SARS-CoV than the bat SARS-related CoV ZC 45. The biological relevance and effects of virulence of the 5′-UTR structures should be investigated further. The 2019-nCoV had various 3′-UTR structures, including BSL, S1, S2, S3, S4, L1, L2, L3, and HVR (Figure 7(D–F)). The 3′-UTR was conserved among 2019-nCoV, human SARS-CoV and SARS-related CoVs [27]. Figure 7.Secondary structure prediction and comparison in the 5′-untranslated region (UTR) and 3′-UTR using the RNAfold WebServer (with minimum free energy and partition function in Fold algorithms and basic options. The SARS 5′- and 3′- UTR was used as a reference to adjust the prediction results.(A) SARS-CoV 5'-UTR; (B) 2019-nCoV (HKU-SZ-005b) 5'-UTR; (C) ZC45 5'-UTR; (D) SARS-CoV 3'-UTR; (E) 2019-nCoV (HKU-SZ-005b) 3'-UTR; (F) ZC45 3'-UTR.

**Figure:**
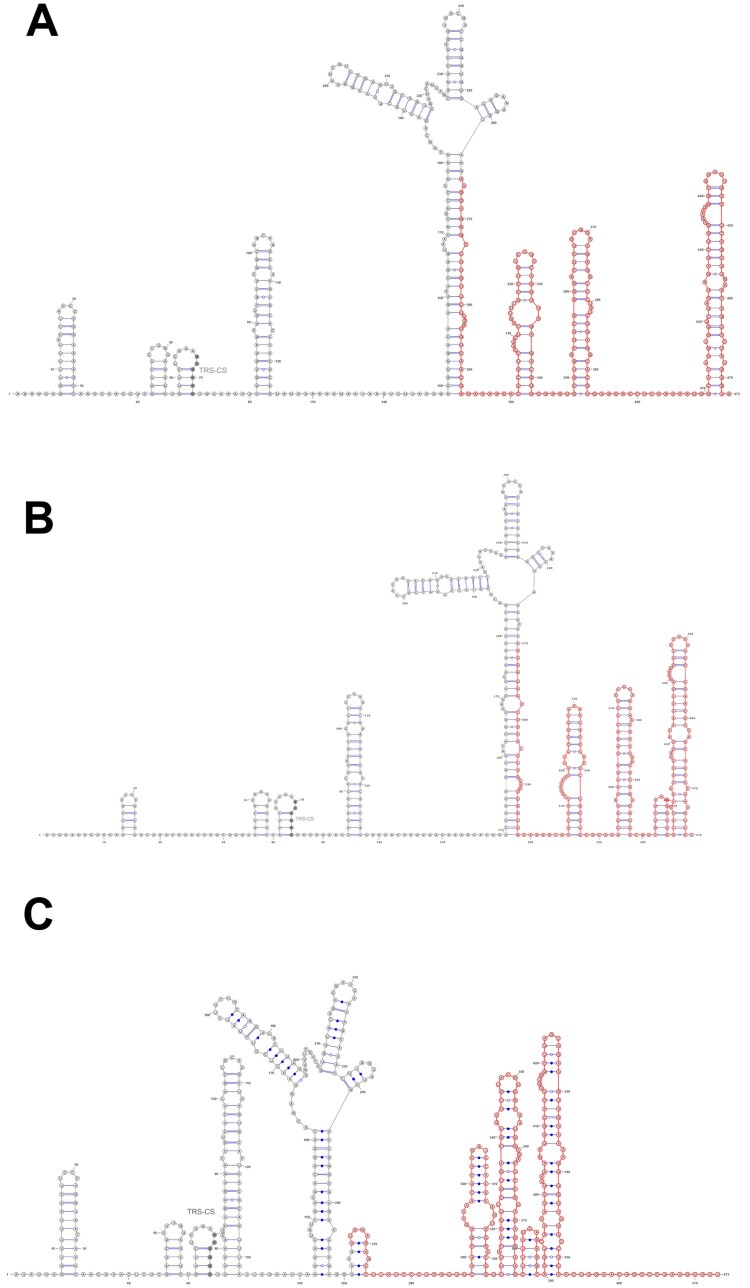

**Figure:**
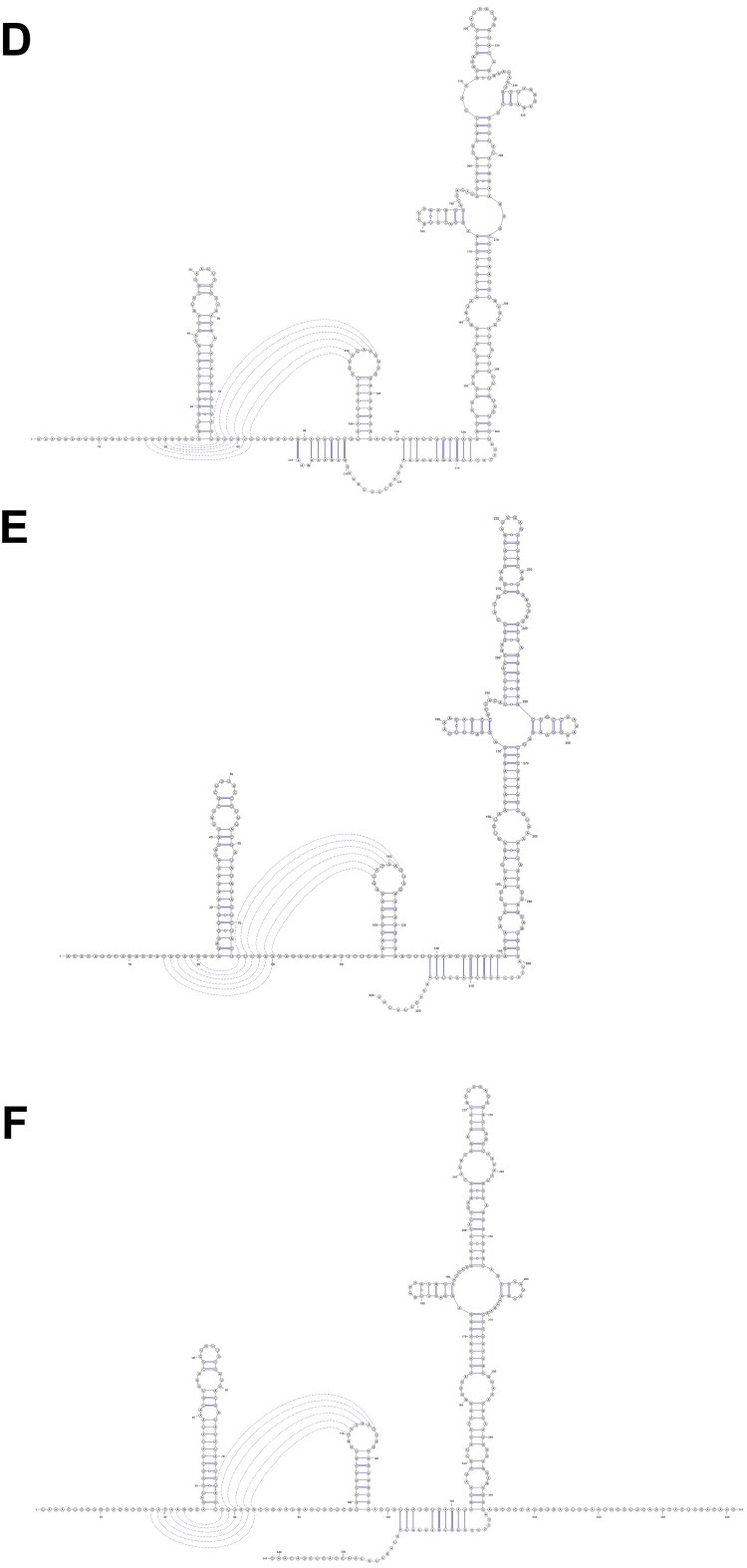

In summary, 2019-nCoV is a novel lineage B *Betacoronavirus* closely related to bat SARS-related coronaviruses. It also has unique genomic features which deserves further investigation to ascertain their roles in viral replication cycle and pathogenesis. More animal sampling to determine its natural animal reservoir and intermediate animal host in the market is important. This will shed light on the evolutionary history of this emerging coronavirus which has jumped into human after the other two zoonotic *Betacoroanviruses*, SARS-CoV and MERS-CoV.
