# Correction

**DOI:** 10.1080/22221751.2020.1737364

**Published:** 2020-03-05

**Article title:** Genomic characterization of the 2019 novel human-pathogenic coronavirus isolated from a patient with atypical pneumonia after visiting Wuhan

**Authors:** Chan, J. F-W., Kok, K-H., Zhu, Z., Chu, H., To K. K-W., Yuan, S., & Yuen, K-Y.

**Journal:** *Emerging Microbes & Infections*

**Bibliometrics:** Volume 9, Number 1, pages 221 –236

**DOI:** https://doi.org/10.1080/22221751.2020.1719902

When the article was first published online, Figure 1 and its caption were wrong. This has now been corrected in online version.

